# Crystal structure and Hirshfeld analysis of diethyl (2*E*,2′*E*)-3,3′-[1-(8-phenyl­isoquinolin-1-yl)-1*H*-indole-2,7-di­yl]diacrylate

**DOI:** 10.1107/S2056989021007829

**Published:** 2021-08-06

**Authors:** Xue-Jun Zhang

**Affiliations:** a Department of Orthopedic Surgery, Zhongda Hospital, School of Medicine, Southeast University, Nanjing 210009, People’s Republic of China

**Keywords:** crystal structure, indole, isoquinolin, weak inter­actions, Hirshfeld analysis

## Abstract

The mol­ecule of title compound, C_33_H_28_N_2_O_4_, comprises an indole unit (*A*), an iso­quinoline moiety (*B*) and a benzene ring (*C*). The dihedral angles between these groups are *A*/*B* = 57.47 (1), *A*/*C* = 18.48 (1) and *B*/*C* = 57.97 (1) °. The ethyl acrylate group at the 2-position is nearly co-planar with the indole unit [3.81 (2)°], while that at the 7-position is distinctly non-coplanar [52.64 (1)°]. Intra­molecular π–π inter­actions between the indole unit and benzene ring help to establish the clip-shaped conformation of the mol­ecule. In the crystal, the mol­ecules are assembled into two-dimensional layers *via* C—H⋯O hydrogen bonds, π–π and C—H⋯π inter­actions. Hirshfeld surface analysis illustrates that the greatest contributions are from H⋯H (63.2%), C⋯H/H⋯C (15.4%) and O⋯H/H⋯O (14.8%) contacts. The terminal C_2_H_5_ group of one of the ethyl acrylate side chains is disordered over two positions of equal occupancy.

## Abstract 2

In the mol­ecule of title compound, intra­molecular π–π inter­actions between the indole unit and benzene ring help to establish the clip-shaped conformation of the mol­ecule. In the crystal, the mol­ecules are assembled into two-dimensional layers *via* C—H⋯O hydrogen bonds, π–π and C—H⋯π inter­actions.

## Chemical context   

As a type of N-containing heterocyclic compound, indoles derivatives are recognized as a privileged structural motif and are widely found in naturally occurring and synthetic mol­ecules with significant biological activity, such as alkaloids, agrochemicals, and drugs (Sharma *et al.*, 2010 ▸; Vargas *et al.*, 2018 ▸). In particular, drugs containing indole subunits exhibit various activities, such as anti-bacterial (Liu, Lauro *et al.*, 2017 ▸), anti-fungal (Xu *et al.*, 2016 ▸), anti-viral (Zhang *et al.*, 2015 ▸), anti-proliferative (Cheng *et al.*, 2019 ▸), anti-inflammatory (Mazzotta *et al.*, 2020 ▸), anti-tumor (Li *et al.*, 2007 ▸), analgesic (Jin *et al.*, 2021 ▸), and a large number of indole-based drugs have been marketed (Mir *et al.*, 2021 ▸; Hussain *et al.*, 2020 ▸), which has made great contributions to human health. Methods for the synthesis of functionalized indoles have therefore attracted a lot of attention over the past few decades. Among them, transition-metal-catalysed direct C—H activation of the indole framework itself has emerged as a fascinating avenue to afford functionalized indole derivatives on account of its atom economy and simplified procedure (Sandtorv, 2015 ▸; Liu, Zhao& Wu, 2017 ▸; Jagtap & Punji, 2020 ▸). On the other hand, because of the much higher reactivity of the 3-position than the 2-position and in turn than the sites in the six-membered ring (Joule *et al.*, 2000 ▸; Fanton *et al.*, 2010 ▸), studies on the synthesis of 2,7-disubstituted indole derivatives have scarcely been reported. Kumar and Sekar employed pyrimidine as a directing group to synthesize 2-acyl indoles and 2,7-di­acyl indoles using a Pd catalyst (Kumar & Sekar, 2015 ▸). Herein, the synthesis, crystal structure and Hirshfeld analysis of the title compound is reported.
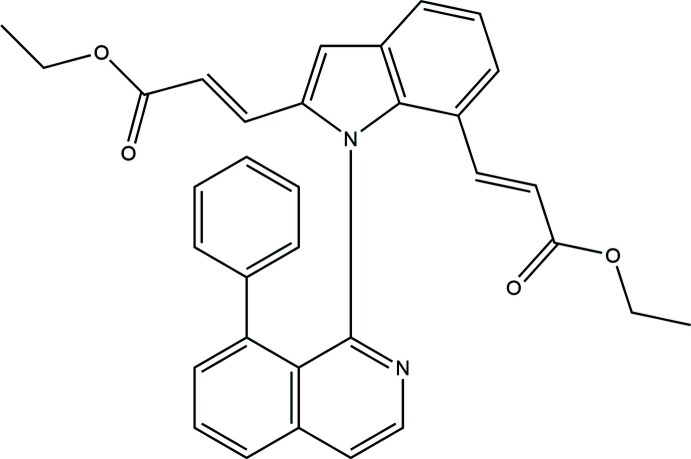


## Structural commentary   

**Figure 1:**
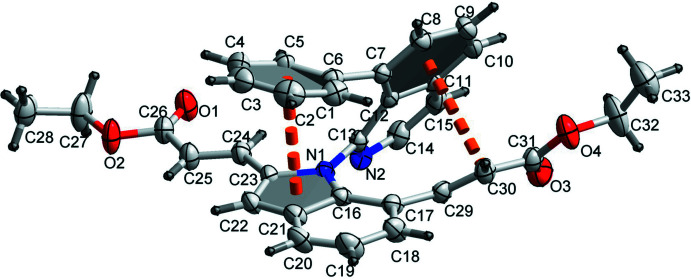
The mol­ecular structure of the title compound with displacement ellipsoids drawn at the 50% probability level showing the intra­molecular π–π and C—H⋯π inter­actions as dashed lines.

The title compound crystallizes in the triclinic *P*-1 space group with one mol­ecule in the asymmetric unit (Fig. 1 ▸). The dihedral angles between the mean plane of the indole unit (*A*, N1/C16/C21–C23), the iso­quinoline moiety (*B*, N2/C7–C15) and the benzene ring (C, C1–C6) are 56.47 (2), 57.97 (1) and 18.48 (1)° for *A*/*B*, *B*/*C* and *A*/*C*, respectively. The benzene ring is almost parallel to the indole unit and hence intra­mol­ecular π–π inter­actions [*Cg*1⋯*Cg*2 = 3.3790 (4) Å, where *Cg*1 and *Cg*2 are the centroids of the N1/C16/C21–C23 and C1–C6 rings, respectively; Fig. 1 ▸] arising from these two aromatic rings were observed, which contribute to the formation of the clip-shaped confirmation. The 2-substituted ethyl acrylate moiety on the indole unit is nearly co-planar with the indole unit [dihedral angle = 3.81 (2)°], while the dihedral angle between the indole unit and the 7-substituted ethyl acrylate moiety is 52.64 (1)°. Further analysis finds that the 7-substituted ethyl acrylate moiety is nearly parallel to the iso­quinoline unit [9.66 (2)°] and thus intra­mol­ecular π–π inter­actions [C30⋯*Cg*3 = 3.3958 (4) Å, *Cg*3 is the centroid of the C7–C12 ring; Fig. 1 ▸] and C—H⋯π inter­actions are observed.

## Supra­molecular features   

**Figure 2:**
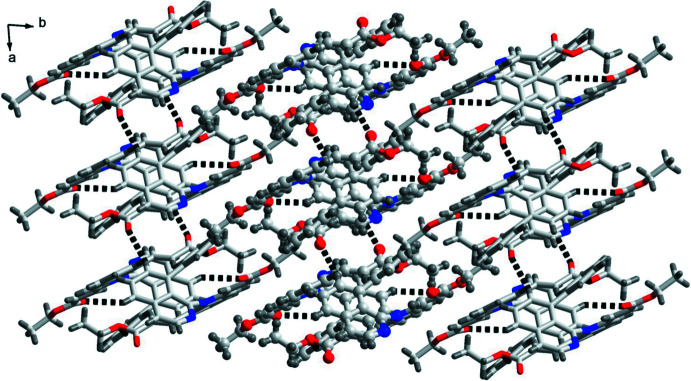
The packing of the title compound showing the two-dimensional layers formed by C—H⋯O hydrogen bonds (dashed lines).

**Figure 3:**
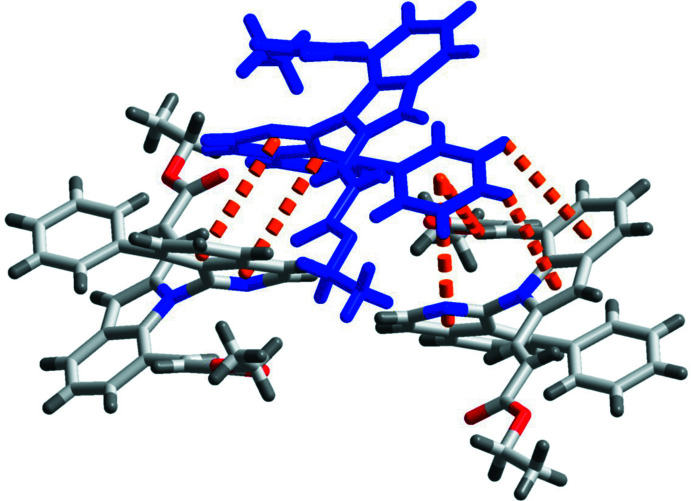
Partial packing diagram of the title compound, showing the π–π and C—H–π inter­actions (red dashed lines).

In the crystal, the mol­ecules are linked by C10—H10*A*⋯O1, C8—H8*A*⋯O3 and C2—H2*A*⋯O4 hydrogen bonds (Fig. 2 ▸, Table 1 ▸), generating two-dimensional layers propagating along the *a*-axis direction. Intermolecular π–π and C—H⋯π inter­actions [3.1990 (5)–4.1187 (6) Å] are observed within the layers (Fig. 3 ▸). The layers are further connected into a three-dimensional network by van der Waals inter­actions.

## Hirshfeld Surface analysis   

**Figure 4:**
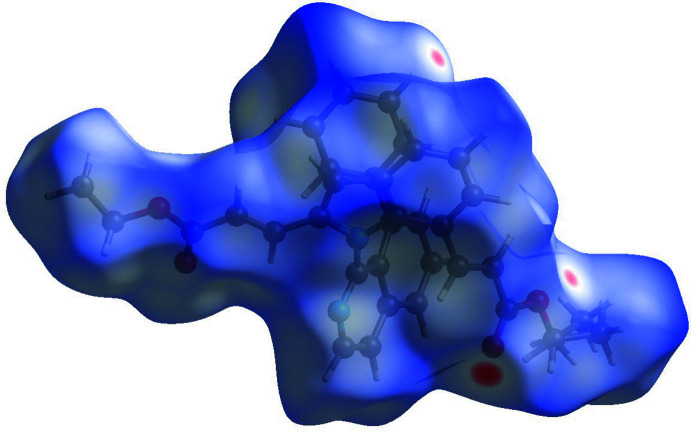
Hirshfeld surfaces of the title compound mapped over *d*_norm_.

**Figure 5:**
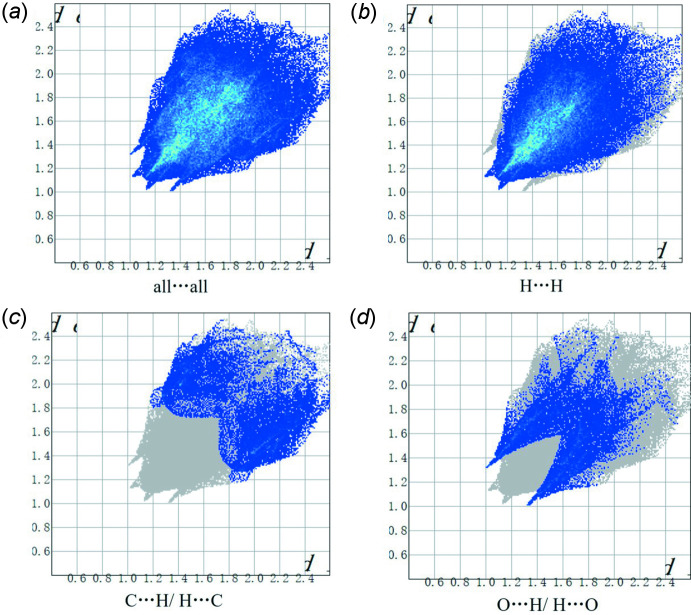
The two-dimensional fingerprint plots for the title compound, showing (*a*) all inter­actions, and delineated into (*b*) H⋯H, (*c*) C⋯H/H⋯C and (*d*) O⋯H/H⋯O inter­actions. The *d*_i_ and *d*_e_ values are the closest inter­nal and external distances (in Å) from given points on the Hirshfeld surface.

A Hirshfeld surface analysis was performed and the associated two-dimensional fingerprint plots were generated using *Crystal Explorer* (Turner *et al.*, 2017 ▸), with a standard resolution of the three-dimensional *d*_norm_ surfaces plotted over a fixed color scale of −0.1861 (red) to 1.7889 (blue) a.u. (Fig. 4 ▸). The red spots symbolize short contacts and negative *d*_norm_ values on the surface correspond to the C—H⋯O hydrogen bonds described above. Two-dimensional fingerprint plots for the H⋯H, H⋯C/C⋯H, and H⋯O/O⋯H contacts are presented in Fig. 5 ▸. At 63.2%, the largest contribution to the overall crystal packing is from H⋯H inter­actions, which are located in the middle region of the fingerprint plot. H⋯C/C⋯H contacts contribute 15.4%, and the H⋯O/O⋯H contacts contribute 14.8% to the Hirshfeld surface, both resulting in a pair of characteristic wings.

## Database survey   

A survey for compounds containing the subunit of the title compound, 2,7-di­vinyl-1*H*-indole, was conducted in the Cambridge Structural Database (Version 5.41, last update November 2019; Groom *et al.*, 2016 ▸). Only one example, namely di­methyl 3,3′-(1-(isoquinolin-1-yl­meth­yl)-1*H*-indole-2,7-di­yl)(2*E*,2′*E*)-diacrylate (XUPXUC; Fanton *et al.*, 2010 ▸), was found, which has a isoquinolin-1-yl­methyl group attached to the nitro­gen atom.

## Synthesis and crystallization   

**Figure 6:**
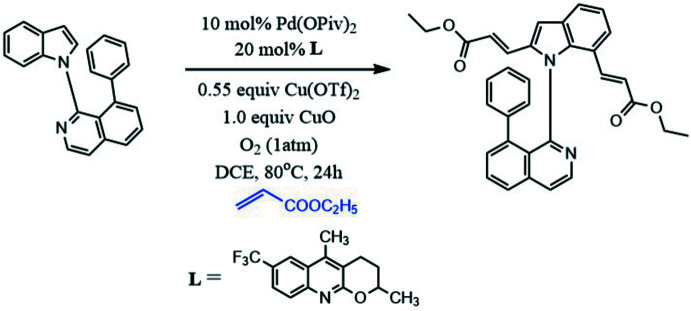
Synthesis of the title compound.

To a 10 mL Schlenk tube was added indole substrate 1-(1*H*-indol-1-yl)-8-phen­yliso­quinoline (0.20 mmol), Pd(OPiv)_2_ (OPiv^−^ = pivalate; 6.2 mg, 10 mol%), *L* [*L* = 2,5-di­methyl-7-(tri­fluoro­meth­yl)-3,4-di­hydro-2*H*-pyrano[2,3-*b*]quinoline; 11.3 mg, 20 mol%], CuO (15.7 mg, 1.0 equiv.) and Cu(OTf)_2_ (OTf^−^ = tri­fluoro­methane­sulfonate; 39.8 mg, 0.55 equiv.) and the tube was purged with O_2_ three times, followed by addition of ethyl acrylate (1.0 mmol) and anhydrous DCE (DCE = 1,2-di­chloro­ethane;1 mL). The formed mixture was stirred at 353 K under Ar for 24 h as monitored by TLC. The solution was then cooled to room temperature, and the solvent was removed under vacuum. The crude product was purified by column chromatography on silica gel to afford the pure product (55% yield). The recrystallization of the title compound in methanol afforded yellow block-shaped crystals. The synthesis is shown in Fig. 6 ▸.

## Refinement   

Crystal data, data collection and structure refinement details are summarized in Table 2 ▸. H atoms were placed in calculated positions (C—H = 0.93–0.97 Å) and refined as riding with *U*_iso_(H) = 1.2*U*_eq_(C) or 1.5*U*_eq_(C-meth­yl). Atoms C32 and C33 were refined as disordered over two partially occupied positions of equal occupancy.

## Supplementary Material

Crystal structure: contains datablock(s) I. DOI: 10.1107/S2056989021007829/zn2008sup1.cif

Structure factors: contains datablock(s) I. DOI: 10.1107/S2056989021007829/zn2008Isup2.hkl

Supporting information file. DOI: 10.1107/S2056989021007829/zn2008Isup3.cml

CCDC reference: 2100362

Additional supporting information:  crystallographic information; 3D view; checkCIF report

## Figures and Tables

**Table 1:** Hydrogen-bond geometry (Å, °) *Cg*3 is the centroid of the N2/C11–C15 ring.

*D*—H⋯*A*	*D*—H	H⋯*A*	*D*⋯*A*	*D*—H⋯*A*
C8—H8*A*⋯O3^i^	0.93	2.49	3.3404 (5)	152
C10—H10*A*⋯O1^ii^	0.93	2.64	3.4252 (5)	143
C2—H2*A*⋯O4^iii^	0.93	2.65	3.5339 (6)	159
C29—H29*A*⋯*Cg*3	0.93	2.86	3.370 (2)	116

Symmetry codes: (i) x-1, y, z; (ii) -x, -y, -z+2; (iii) -x, -y, -z+1.

**Table 2:** Experimental details

Crystal data	
Chemical formula	C_33_H_28_N_2_O_4_
*M*_r_	516.57
Crystal system, space group	Triclinic, *P*\overline{1}
Temperature (K)	296
*a*, *b*, *c* (Å)	7.6918 (11), 13.299 (2), 14.130 (2)
α, β, γ (°)	75.026 (2), 81.728 (3), 79.838 (2)
*V* (Å^3^)	1367.1 (4)
*Z*	2
Radiation type	Mo *K*α
μ (mm^−1^)	0.08
Crystal size (mm)	0.25 × 0.22 × 0.18

Data collection	
Diffractometer	Bruker APEXII CCD
Absorption correction	Multi-scan (*SADABS*; Krause *et al.*, 2015 ▸)
*T*_min_, *T*_max_	0.980, 0.985
No. of measured, independent and observed [*I* > 2σ(*I*)] reflections	7633, 4787, 3595
*R*_int_	0.019
(sin θ/λ)_max_ (Å^−1^)	0.595

Refinement	
*R*[*F*^2^ > 2σ(*F* ^2^)], *wR*(*F* ^2^), *S*	0.042, 0.118, 1.02
No. of reflections	4787
No. of parameters	361
No. of restraints	12
H-atom treatment	H atoms treated by a mixture of independent and constrained refinement
Δρ_max_, Δρ_min_ (e Å^−3^)	0.19, −0.17

Computer programs: *APEX2* and *SAINT* (Bruker, 2014 ▸), *SHELXS97*, *SHELXL97* and *SHELXTL* (Sheldrick, 2008 ▸).

## supplementary crystallographic information

### Crystal data

**Table:**

C_33_H_28_N_2_O_4_	*Z* = 2
*M**_r_* = 516.57	*F*(000) = 544
Triclinic, *P*1	*D*_x_ = 1.255 Mg m^−^^3^
*a* = 7.6918 (11) Å	Mo *K*α radiation, λ = 0.71073 Å
*b* = 13.299 (2) Å	Cell parameters from 2873 reflections
*c* = 14.130 (2) Å	θ = 2.4–26.8°
α = 75.026 (2)°	µ = 0.08 mm^−^^1^
β = 81.728 (3)°	*T* = 296 K
γ = 79.838 (2)°	Block, yellow
*V* = 1367.1 (4) Å^3^	0.25 × 0.22 × 0.18 mm

### Data collection

**Table:**

Bruker APEXII CCD diffractometer	4787 independent reflections
Radiation source: fine-focus sealed tube	3595 reflections with *I* > 2σ(*I*)
Graphite monochromator	*R*_int_ = 0.019
φ and ω scans	θ_max_ = 25.0°, θ_min_ = 1.6°
Absorption correction: multi-scan (SADABS; Krause *et al.*, 2015)	*h* = −8→9
*T*_min_ = 0.980, *T*_max_ = 0.985	*k* = −15→15
7633 measured reflections	*l* = −16→14

### Refinement

**Table:**

Refinement on *F*^2^	Primary atom site location: structure-invariant direct methods
Least-squares matrix: full	Secondary atom site location: difference Fourier map
*R*[*F*^2^ > 2σ(*F*^2^)] = 0.042	Hydrogen site location: inferred from neighbouring sites
*wR*(*F*^2^) = 0.118	H atoms treated by a mixture of independent and constrained refinement
*S* = 1.02	*w* = 1/[σ^2^(*F*_o_^2^) + (0.0501*P*)^2^ + 0.2961*P*] where *P* = (*F*_o_^2^ + 2*F*_c_^2^)/3
4787 reflections	(Δ/σ)_max_ < 0.001
361 parameters	Δρ_max_ = 0.19 e Å^−^^3^
12 restraints	Δρ_min_ = −0.17 e Å^−^^3^

### Special details

**Table:**

Geometry. All esds (except the esd in the dihedral angle between two l.s. planes) are estimated using the full covariance matrix. The cell esds are taken into account individually in the estimation of esds in distances, angles and torsion angles; correlations between esds in cell parameters are only used when they are defined by crystal symmetry. An approximate (isotropic) treatment of cell esds is used for estimating esds involving l.s. planes.
Refinement. Refinement of F^2^ against ALL reflections. The weighted R-factor wR and goodness of fit S are based on F^2^, conventional R-factors R are based on F, with F set to zero for negative F^2^. The threshold expression of F^2^ > 2sigma(F^2^) is used only for calculating R-factors(gt) etc. and is not relevant to the choice of reflections for refinement. R-factors based on F^2^ are statistically about twice as large as those based on F, and R- factors based on ALL data will be even larger.

### Fractional atomic coordinates and isotropic or equivalent isotropic displacement parameters (Å^2^)

**Table:**

	*x*	*y*	*z*	*U*_iso_*/*U*_eq_	Occ. (<1)
C1	−0.2667 (3)	0.12835 (16)	0.62659 (15)	0.0669 (5)	
H1A	−0.2063	0.0785	0.5933	0.080*	
C2	−0.3886 (3)	0.2076 (2)	0.58064 (17)	0.0805 (6)	
H2A	−0.4085	0.2117	0.5163	0.097*	
C3	−0.4814 (3)	0.28071 (18)	0.62914 (18)	0.0781 (6)	
H3A	−0.5642	0.3342	0.5979	0.094*	
C4	−0.4512 (3)	0.27430 (16)	0.72388 (17)	0.0714 (6)	
H4A	−0.5149	0.3231	0.7573	0.086*	
C5	−0.3266 (2)	0.19584 (14)	0.77003 (14)	0.0579 (5)	
H5A	−0.3061	0.1929	0.8340	0.070*	
C6	−0.2320 (2)	0.12148 (13)	0.72222 (13)	0.0492 (4)	
C7	−0.1090 (2)	0.03181 (12)	0.77462 (12)	0.0486 (4)	
C8	−0.1445 (3)	−0.06862 (14)	0.78380 (15)	0.0645 (5)	
H8A	−0.2356	−0.0776	0.7514	0.077*	
C9	−0.0479 (3)	−0.15732 (15)	0.84018 (17)	0.0737 (6)	
H9A	−0.0704	−0.2240	0.8409	0.088*	
C10	0.0779 (3)	−0.14745 (14)	0.89381 (15)	0.0668 (5)	
H10A	0.1360	−0.2067	0.9343	0.080*	
C11	0.1205 (2)	−0.04680 (13)	0.88805 (13)	0.0530 (4)	
C12	0.0358 (2)	0.04292 (12)	0.82278 (11)	0.0439 (4)	
C13	0.1124 (2)	0.13686 (12)	0.80938 (12)	0.0444 (4)	
C14	0.2831 (3)	0.06566 (16)	0.93629 (15)	0.0661 (5)	
H14A	0.3522	0.0754	0.9812	0.079*	
C15	0.2454 (3)	−0.03174 (16)	0.94468 (14)	0.0644 (5)	
H15A	0.3022	−0.0890	0.9880	0.077*	
C16	0.1046 (2)	0.21406 (12)	0.62857 (12)	0.0475 (4)	
C17	0.1905 (2)	0.13020 (13)	0.58706 (13)	0.0529 (4)	
C18	0.1870 (3)	0.14647 (16)	0.48587 (14)	0.0706 (6)	
H18A	0.2414	0.0930	0.4556	0.085*	
C19	0.1055 (3)	0.23942 (17)	0.42822 (15)	0.0809 (7)	
H19A	0.1028	0.2456	0.3614	0.097*	
C20	0.0292 (3)	0.32200 (16)	0.46865 (15)	0.0734 (6)	
H20A	−0.0221	0.3846	0.4294	0.088*	
C21	0.0296 (2)	0.31074 (13)	0.57007 (13)	0.0556 (4)	
C22	−0.0357 (3)	0.37794 (13)	0.63444 (14)	0.0596 (5)	
H22A	−0.0906	0.4472	0.6159	0.072*	
C23	−0.0048 (2)	0.32418 (12)	0.72834 (13)	0.0513 (4)	
C24	−0.0523 (2)	0.35547 (13)	0.82037 (14)	0.0559 (4)	
H24A	−0.0273	0.3047	0.8777	0.067*	
C25	−0.1282 (3)	0.44978 (15)	0.83035 (15)	0.0655 (5)	
H25A	−0.1514	0.5028	0.7743	0.079*	
C26	−0.1769 (3)	0.47315 (14)	0.92742 (16)	0.0620 (5)	
C27	−0.3135 (4)	0.60395 (19)	1.01189 (17)	0.0965 (8)	
H27A	−0.3689	0.5494	1.0600	0.116*	
H27B	−0.2107	0.6153	1.0382	0.116*	
C28	−0.4373 (3)	0.69986 (18)	0.99332 (18)	0.0925 (8)	
H28A	−0.4743	0.7218	1.0536	0.139*	
H28B	−0.5390	0.6881	0.9677	0.139*	
H28C	−0.3813	0.7537	0.9462	0.139*	
C29	0.2884 (2)	0.03339 (13)	0.64329 (13)	0.0532 (4)	
H29A	0.3565	0.0398	0.6901	0.064*	
C30	0.2869 (3)	−0.06246 (14)	0.63238 (14)	0.0587 (5)	
H30A	0.2193	−0.0711	0.5863	0.070*	
C31	0.3893 (3)	−0.15528 (14)	0.69115 (14)	0.0594 (5)	
C32	0.4348 (4)	−0.34173 (18)	0.7337 (2)	0.1125 (10)	0.50
H32A	0.4220	−0.3423	0.8031	0.135*	0.50
H32B	0.5603	−0.3516	0.7109	0.135*	0.50
C33	0.3476 (11)	−0.4261 (6)	0.7177 (6)	0.122 (3)	0.50
H33A	0.4013	−0.4931	0.7538	0.183*	0.50
H33B	0.3616	−0.4246	0.6488	0.183*	0.50
H33C	0.2235	−0.4150	0.7402	0.183*	0.50
C32'	0.4348 (4)	−0.34173 (18)	0.7337 (2)	0.1125 (10)	0.50
H32C	0.5485	−0.3315	0.7489	0.135*	0.50
H32D	0.3629	−0.3649	0.7952	0.135*	0.50
C33'	0.4613 (11)	−0.4205 (6)	0.6780 (6)	0.123 (3)	0.50
H33D	0.5198	−0.4853	0.7155	0.185*	0.50
H33E	0.5333	−0.3977	0.6174	0.185*	0.50
H33F	0.3484	−0.4312	0.6639	0.185*	0.50
N1	0.07802 (17)	0.22204 (9)	0.72568 (10)	0.0450 (3)	
N2	0.22465 (19)	0.15007 (11)	0.86518 (11)	0.0565 (4)	
O1	−0.1490 (2)	0.41273 (11)	1.00476 (11)	0.0842 (5)	
O2	−0.2587 (2)	0.57132 (11)	0.92002 (10)	0.0829 (5)	
O3	0.4949 (2)	−0.15455 (12)	0.74563 (12)	0.0822 (4)	
O4	0.3472 (2)	−0.24346 (10)	0.67786 (11)	0.0831 (5)	

### Atomic displacement parameters (Å^2^)

**Table:**

	*U*^11^	*U*^22^	*U*^33^	*U*^12^	*U*^13^	*U*^23^
C1	0.0716 (13)	0.0773 (13)	0.0607 (12)	−0.0157 (10)	−0.0119 (10)	−0.0266 (10)
C2	0.0833 (15)	0.0984 (17)	0.0651 (14)	−0.0211 (13)	−0.0271 (12)	−0.0129 (13)
C3	0.0634 (13)	0.0787 (15)	0.0846 (16)	−0.0108 (11)	−0.0249 (12)	0.0038 (12)
C4	0.0633 (12)	0.0652 (12)	0.0771 (15)	0.0021 (10)	−0.0026 (11)	−0.0120 (11)
C5	0.0602 (11)	0.0592 (11)	0.0508 (10)	−0.0031 (9)	−0.0021 (9)	−0.0124 (8)
C6	0.0483 (9)	0.0527 (10)	0.0500 (10)	−0.0156 (7)	−0.0014 (8)	−0.0147 (8)
C7	0.0525 (10)	0.0448 (9)	0.0491 (10)	−0.0095 (7)	0.0042 (8)	−0.0158 (7)
C8	0.0677 (12)	0.0551 (11)	0.0759 (13)	−0.0193 (9)	0.0030 (10)	−0.0234 (10)
C9	0.0843 (15)	0.0413 (10)	0.0905 (16)	−0.0173 (10)	0.0150 (13)	−0.0148 (10)
C10	0.0763 (13)	0.0437 (10)	0.0669 (13)	0.0004 (9)	0.0090 (11)	−0.0042 (9)
C11	0.0543 (10)	0.0480 (10)	0.0489 (10)	0.0021 (8)	0.0057 (8)	−0.0103 (8)
C12	0.0480 (9)	0.0402 (8)	0.0418 (9)	−0.0028 (7)	0.0040 (7)	−0.0136 (7)
C13	0.0468 (9)	0.0431 (9)	0.0437 (9)	−0.0007 (7)	−0.0024 (7)	−0.0164 (7)
C14	0.0640 (12)	0.0750 (14)	0.0611 (12)	0.0068 (10)	−0.0208 (10)	−0.0231 (10)
C15	0.0640 (12)	0.0625 (12)	0.0553 (11)	0.0132 (9)	−0.0079 (9)	−0.0077 (9)
C16	0.0547 (10)	0.0426 (9)	0.0473 (10)	−0.0154 (7)	−0.0011 (8)	−0.0113 (7)
C17	0.0621 (11)	0.0493 (10)	0.0500 (10)	−0.0155 (8)	0.0041 (8)	−0.0170 (8)
C18	0.1023 (16)	0.0597 (12)	0.0512 (12)	−0.0169 (11)	0.0054 (11)	−0.0193 (9)
C19	0.130 (2)	0.0674 (13)	0.0451 (11)	−0.0225 (13)	−0.0057 (12)	−0.0101 (10)
C20	0.1067 (17)	0.0536 (11)	0.0566 (12)	−0.0204 (11)	−0.0122 (11)	0.0007 (9)
C21	0.0686 (11)	0.0449 (9)	0.0539 (11)	−0.0169 (8)	−0.0061 (9)	−0.0073 (8)
C22	0.0717 (12)	0.0369 (9)	0.0686 (13)	−0.0089 (8)	−0.0076 (10)	−0.0090 (9)
C23	0.0579 (10)	0.0372 (9)	0.0603 (11)	−0.0068 (7)	−0.0038 (8)	−0.0156 (8)
C24	0.0626 (11)	0.0440 (9)	0.0637 (11)	−0.0046 (8)	−0.0060 (9)	−0.0198 (8)
C25	0.0793 (13)	0.0527 (11)	0.0630 (12)	0.0064 (9)	−0.0089 (10)	−0.0213 (9)
C26	0.0675 (12)	0.0489 (11)	0.0704 (13)	0.0050 (9)	−0.0107 (10)	−0.0226 (10)
C27	0.129 (2)	0.0874 (16)	0.0712 (15)	0.0354 (15)	−0.0210 (14)	−0.0450 (13)
C28	0.1016 (18)	0.0868 (16)	0.0880 (17)	0.0128 (13)	0.0026 (14)	−0.0434 (14)
C29	0.0549 (10)	0.0556 (10)	0.0498 (10)	−0.0072 (8)	0.0066 (8)	−0.0209 (8)
C30	0.0711 (12)	0.0542 (11)	0.0530 (11)	−0.0030 (9)	−0.0060 (9)	−0.0211 (8)
C31	0.0649 (12)	0.0581 (11)	0.0565 (11)	−0.0013 (9)	−0.0013 (9)	−0.0238 (9)
C32	0.169 (3)	0.0536 (14)	0.114 (2)	0.0120 (15)	−0.062 (2)	−0.0116 (14)
C33	0.171 (6)	0.063 (3)	0.126 (5)	−0.012 (4)	−0.035 (5)	−0.007 (3)
C32'	0.169 (3)	0.0536 (14)	0.114 (2)	0.0120 (15)	−0.062 (2)	−0.0116 (14)
C33'	0.151 (6)	0.068 (4)	0.152 (6)	0.019 (4)	−0.049 (5)	−0.035 (4)
N1	0.0526 (8)	0.0371 (7)	0.0476 (8)	−0.0073 (6)	−0.0034 (6)	−0.0147 (6)
N2	0.0544 (9)	0.0595 (9)	0.0605 (9)	−0.0024 (7)	−0.0134 (7)	−0.0229 (8)
O1	0.1208 (13)	0.0566 (8)	0.0690 (10)	0.0082 (8)	−0.0098 (9)	−0.0183 (7)
O2	0.1138 (12)	0.0630 (9)	0.0678 (9)	0.0313 (8)	−0.0199 (8)	−0.0324 (7)
O3	0.0754 (10)	0.0800 (10)	0.0964 (12)	−0.0037 (7)	−0.0272 (9)	−0.0254 (8)
O4	0.1246 (13)	0.0488 (8)	0.0808 (10)	0.0030 (8)	−0.0383 (9)	−0.0200 (7)

### Geometric parameters (Å, º)

**Table:**

C1—C2	1.373 (3)	C19—H19A	0.9300
C1—C6	1.392 (2)	C20—C21	1.402 (3)
C1—H1A	0.9300	C20—H20A	0.9300
C2—C3	1.373 (3)	C21—C22	1.417 (3)
C2—H2A	0.9300	C22—C23	1.367 (2)
C3—C4	1.370 (3)	C22—H22A	0.9300
C3—H3A	0.9300	C23—N1	1.402 (2)
C4—C5	1.381 (3)	C23—C24	1.445 (2)
C4—H4A	0.9300	C24—C25	1.320 (2)
C5—C6	1.383 (2)	C24—H24A	0.9300
C5—H5A	0.9300	C25—C26	1.466 (3)
C6—C7	1.488 (2)	C25—H25A	0.9300
C7—C8	1.380 (2)	C26—O1	1.200 (2)
C7—C12	1.433 (2)	C26—O2	1.330 (2)
C8—C9	1.398 (3)	C27—C28	1.440 (3)
C8—H8A	0.9300	C27—O2	1.456 (2)
C9—C10	1.355 (3)	C27—H27A	0.9700
C9—H9A	0.9300	C27—H27B	0.9700
C10—C11	1.413 (3)	C28—H28A	0.9600
C10—H10A	0.9300	C28—H28B	0.9600
C11—C15	1.406 (3)	C28—H28C	0.9600
C11—C12	1.424 (2)	C29—C30	1.325 (2)
C12—C13	1.431 (2)	C29—H29A	0.9300
C13—N2	1.311 (2)	C30—C31	1.466 (3)
C13—N1	1.430 (2)	C30—H30A	0.9300
C14—C15	1.349 (3)	C31—O3	1.200 (2)
C14—N2	1.357 (2)	C31—O4	1.335 (2)
C14—H14A	0.9300	C32—O4	1.447 (3)
C15—H15A	0.9300	C32—C33	1.483 (8)
C16—N1	1.387 (2)	C32—H32A	0.9700
C16—C17	1.411 (2)	C32—H32B	0.9700
C16—C21	1.413 (2)	C33—H33A	0.9600
C17—C18	1.393 (3)	C33—H33B	0.9600
C17—C29	1.469 (2)	C33—H33C	0.9600
C18—C19	1.392 (3)	C33'—H33D	0.9600
C18—H18A	0.9300	C33'—H33E	0.9600
C19—C20	1.370 (3)	C33'—H33F	0.9600
			
C2—C1—C6	121.0 (2)	C20—C21—C16	119.45 (17)
C2—C1—H1A	119.5	C20—C21—C22	133.75 (18)
C6—C1—H1A	119.5	C16—C21—C22	106.78 (16)
C1—C2—C3	120.4 (2)	C23—C22—C21	108.75 (15)
C1—C2—H2A	119.8	C23—C22—H22A	125.6
C3—C2—H2A	119.8	C21—C22—H22A	125.6
C4—C3—C2	119.5 (2)	C22—C23—N1	108.15 (15)
C4—C3—H3A	120.3	C22—C23—C24	130.46 (16)
C2—C3—H3A	120.3	N1—C23—C24	121.29 (15)
C3—C4—C5	120.4 (2)	C25—C24—C23	125.78 (18)
C3—C4—H4A	119.8	C25—C24—H24A	117.1
C5—C4—H4A	119.8	C23—C24—H24A	117.1
C4—C5—C6	120.80 (18)	C24—C25—C26	121.80 (19)
C4—C5—H5A	119.6	C24—C25—H25A	119.1
C6—C5—H5A	119.6	C26—C25—H25A	119.1
C5—C6—C1	117.88 (17)	O1—C26—O2	123.11 (18)
C5—C6—C7	120.82 (16)	O1—C26—C25	125.45 (17)
C1—C6—C7	121.08 (16)	O2—C26—C25	111.44 (17)
C8—C7—C12	117.69 (16)	C28—C27—O2	109.01 (19)
C8—C7—C6	117.81 (16)	C28—C27—H27A	109.9
C12—C7—C6	124.32 (14)	O2—C27—H27A	109.9
C7—C8—C9	122.14 (19)	C28—C27—H27B	109.9
C7—C8—H8A	118.9	O2—C27—H27B	109.9
C9—C8—H8A	118.9	H27A—C27—H27B	108.3
C10—C9—C8	120.86 (18)	C27—C28—H28A	109.5
C10—C9—H9A	119.6	C27—C28—H28B	109.5
C8—C9—H9A	119.6	H28A—C28—H28B	109.5
C9—C10—C11	119.79 (18)	C27—C28—H28C	109.5
C9—C10—H10A	120.1	H28A—C28—H28C	109.5
C11—C10—H10A	120.1	H28B—C28—H28C	109.5
C15—C11—C10	122.10 (17)	C30—C29—C17	124.97 (17)
C15—C11—C12	118.29 (16)	C30—C29—H29A	117.5
C10—C11—C12	119.61 (18)	C17—C29—H29A	117.5
C11—C12—C13	114.52 (15)	C29—C30—C31	121.64 (18)
C11—C12—C7	119.21 (14)	C29—C30—H30A	119.2
C13—C12—C7	126.23 (14)	C31—C30—H30A	119.2
N2—C13—N1	115.16 (14)	O3—C31—O4	123.31 (18)
N2—C13—C12	125.01 (15)	O3—C31—C30	125.94 (18)
N1—C13—C12	119.66 (14)	O4—C31—C30	110.74 (17)
C15—C14—N2	122.84 (18)	O4—C32—C33	106.4 (4)
C15—C14—H14A	118.6	O4—C32—H32A	110.4
N2—C14—H14A	118.6	C33—C32—H32A	110.4
C14—C15—C11	120.14 (17)	O4—C32—H32B	110.4
C14—C15—H15A	119.9	C33—C32—H32B	110.4
C11—C15—H15A	119.9	H32A—C32—H32B	108.6
N1—C16—C17	130.45 (15)	C32—C33—H33A	109.5
N1—C16—C21	107.63 (14)	C32—C33—H33B	109.5
C17—C16—C21	121.91 (16)	H33A—C33—H33B	109.5
C18—C17—C16	115.80 (17)	C32—C33—H33C	109.5
C18—C17—C29	120.48 (16)	H33A—C33—H33C	109.5
C16—C17—C29	123.62 (16)	H33B—C33—H33C	109.5
C19—C18—C17	122.75 (18)	H33D—C33'—H33E	109.5
C19—C18—H18A	118.6	H33D—C33'—H33F	109.5
C17—C18—H18A	118.6	H33E—C33'—H33F	109.5
C20—C19—C18	120.90 (19)	C16—N1—C23	108.62 (13)
C20—C19—H19A	119.6	C16—N1—C13	125.19 (12)
C18—C19—H19A	119.6	C23—N1—C13	125.88 (13)
C19—C20—C21	119.03 (19)	C13—N2—C14	117.40 (16)
C19—C20—H20A	120.5	C26—O2—C27	116.55 (16)
C21—C20—H20A	120.5	C31—O4—C32	116.81 (18)
			
C6—C1—C2—C3	−1.2 (3)	C19—C20—C21—C16	−1.5 (3)
C1—C2—C3—C4	0.2 (3)	C19—C20—C21—C22	−179.9 (2)
C2—C3—C4—C5	0.9 (3)	N1—C16—C21—C20	−176.43 (16)
C3—C4—C5—C6	−0.9 (3)	C17—C16—C21—C20	4.4 (3)
C4—C5—C6—C1	−0.1 (3)	N1—C16—C21—C22	2.38 (19)
C4—C5—C6—C7	−174.84 (16)	C17—C16—C21—C22	−176.77 (16)
C2—C1—C6—C5	1.2 (3)	C20—C21—C22—C23	177.6 (2)
C2—C1—C6—C7	175.87 (17)	C16—C21—C22—C23	−1.0 (2)
C5—C6—C7—C8	118.61 (19)	C21—C22—C23—N1	−0.8 (2)
C1—C6—C7—C8	−56.0 (2)	C21—C22—C23—C24	−176.96 (18)
C5—C6—C7—C12	−56.3 (2)	C22—C23—C24—C25	−5.2 (3)
C1—C6—C7—C12	129.10 (18)	N1—C23—C24—C25	179.04 (18)
C12—C7—C8—C9	1.9 (3)	C23—C24—C25—C26	177.95 (18)
C6—C7—C8—C9	−173.33 (17)	C24—C25—C26—O1	2.5 (3)
C7—C8—C9—C10	4.4 (3)	C24—C25—C26—O2	−177.10 (18)
C8—C9—C10—C11	−4.1 (3)	C18—C17—C29—C30	−43.1 (3)
C9—C10—C11—C15	177.73 (18)	C16—C17—C29—C30	140.80 (19)
C9—C10—C11—C12	−2.6 (3)	C17—C29—C30—C31	179.64 (16)
C15—C11—C12—C13	10.8 (2)	C29—C30—C31—O3	−7.9 (3)
C10—C11—C12—C13	−168.87 (15)	C29—C30—C31—O4	170.90 (17)
C15—C11—C12—C7	−171.45 (15)	C17—C16—N1—C23	176.17 (17)
C10—C11—C12—C7	8.8 (2)	C21—C16—N1—C23	−2.89 (18)
C8—C7—C12—C11	−8.4 (2)	C17—C16—N1—C13	−9.9 (3)
C6—C7—C12—C11	166.54 (15)	C21—C16—N1—C13	171.01 (14)
C8—C7—C12—C13	169.00 (16)	C22—C23—N1—C16	2.29 (19)
C6—C7—C12—C13	−16.1 (2)	C24—C23—N1—C16	178.89 (15)
C11—C12—C13—N2	−14.1 (2)	C22—C23—N1—C13	−171.56 (15)
C7—C12—C13—N2	168.39 (15)	C24—C23—N1—C13	5.0 (2)
C11—C12—C13—N1	161.00 (13)	N2—C13—N1—C16	122.36 (16)
C7—C12—C13—N1	−16.5 (2)	C12—C13—N1—C16	−53.2 (2)
N2—C14—C15—C11	−9.5 (3)	N2—C13—N1—C23	−64.8 (2)
C10—C11—C15—C14	179.47 (17)	C12—C13—N1—C23	119.66 (17)
C12—C11—C15—C14	−0.2 (3)	N1—C13—N2—C14	−169.93 (14)
N1—C16—C17—C18	177.25 (17)	C12—C13—N2—C14	5.4 (2)
C21—C16—C17—C18	−3.8 (2)	C15—C14—N2—C13	7.0 (3)
N1—C16—C17—C29	−6.5 (3)	O1—C26—O2—C27	0.5 (3)
C21—C16—C17—C29	172.48 (16)	C25—C26—O2—C27	−179.9 (2)
C16—C17—C18—C19	0.4 (3)	C28—C27—O2—C26	−165.3 (2)
C29—C17—C18—C19	−175.98 (19)	O3—C31—O4—C32	0.5 (3)
C17—C18—C19—C20	2.4 (3)	C30—C31—O4—C32	−178.3 (2)
C18—C19—C20—C21	−1.8 (3)	C33—C32—O4—C31	171.9 (4)

### Hydrogen-bond geometry (Å, º)

*Cg*3 is the centroid of the N2/C11–C15 ring.

**Table:**

*D*—H···*A*	*D*—H	H···*A*	*D*···*A*	*D*—H···*A*
C8—H8*A*···O3^i^	0.93	2.49	3.3404 (5)	152
C10—H10*A*···O1^ii^	0.93	2.64	3.4252 (5)	143
C2—H2*A*···O4^iii^	0.93	2.65	3.5339 (6)	159
C29—H29*A*···*Cg*3	0.93	2.86	3.370 (2)	116

Symmetry codes: (i) *x*−1, *y*, *z*; (ii) −*x*, −*y*, −*z*+2; (iii) −*x*, −*y*, −*z*+1.
